# Positive allosteric modulation of the adenosine A_2a_ receptor attenuates inflammation

**DOI:** 10.1186/s12950-014-0037-0

**Published:** 2014-11-28

**Authors:** Ajith A Welihinda, Edward P Amento

**Affiliations:** Molecular Medicine Research Institute, 428 Oakmead Parkway, Sunnyvale, CA 94085 USA

**Keywords:** Adenosine A_2a_ receptor, G protein-couple receptor, Allosteric enhancement, Inflammation, Positive allosteric modulator, Inhibition of TNF-α production

## Abstract

**Background:**

Adenosine is produced at high levels at inflamed sites as a by-product of cellular activation and breakdown. Adenosine mediates its anti-inflammatory activity primarily through the adenosine A_2a_ receptor (A_2a_R), a member of the G-protein coupled receptors. A_2a_R agonists have demonstrated anti-inflammatory efficacy, however, their therapeutic utility is hindered by a lack of adenosine receptor subtype selectivity upon systemic exposure. We sought to harness the anti-inflammatory effects of adenosine by enhancing the responsiveness of A_2a_R to endogenously produced adenosine through allosteric modulation. We have identified a family of positive allosteric modulators (PAMs) of the A_2a_R. Using one member of this PAM family, AEA061, we demonstrate that A_2a_Rs are amenable to allosteric enhancement and such enhancement produces increased A_2a_R signaling and diminished inflammation in vivo.

**Methods:**

A_2a_R activity was evaluated using a cell-based cAMP assay. Binding affinity of A_2a_R was determined using [^3^H]CGS 21680. A_2a_R-mediated G-protein activation was quantified using [^35^S]GTP-γS. The effect of AEA061 on cytokine production was evaluated using primary monocytes and splenocytes. The anti-inflammatory effect of AEA061 was evaluated in the LPS-induced mouse model of inflammation.

**Results:**

AEA061 had no detectable intrinsic agonist activity towards either rat or human A_2a_Rs. AEA061 enhanced the efficacy of adenosine to rat and human A_2a_Rs by 11.5 and 2.8 fold respectively. AEA061 also enhanced the maximal response by 4.2 and 2.1 fold for the rat and the human A_2a_R respectively. AEA061 potentiated agonist-mediated Gα activation by 3.7 fold. Additionally, AEA061 enhanced both the affinity as well as the B_max_ at the human A_2a_R by 1.8 and 3 fold respectively. Consistent with the anti-inflammatory role of the A_2a_R, allosteric enhancement with AEA061 inhibited the production of TNF-α, MIP-1α, MIP-1β, MIP-2, IL-1α, KC and RANTES by LPS-stimulated macrophages and/or splenocytes. Moreover, AEA061 reduced circulating plasma TNF-α and MCP-1 levels and increased plasma IL-10 in endotoxemic A_2a_R intact, but not in A_2a_R deficient, mice.

**Conclusions:**

AEA061 increases affinity and B_max_ of A_2a_R to adenosine, thereby increasing adenosine potency and efficacy, which translates to enhanced A_2a_R responsiveness. Since the A_2a_R negatively regulates inflammation, PAMs of the receptor offer a novel means of modulating inflammatory processes.

## Background

Inflamed/injured tissues produce pro-inflammatory cytokines such as tumor necrosis factor-alpha (TNF-α) to mediate cellular processes required to remove injurious stimuli and initiate the healing process that is essential for survival of the organism. Cells utilize a variety of receptors and signaling pathways to activate pro-inflammatory cytokine production. For example, engagement of the pathogen-associated pattern recognition family of receptors (TLR; [1]) as well as antigen-dependent ligation of Fcγ receptors (FcγR; [2]) promotes the production of pro-inflammatory cytokines. However, if unchecked, chronic inflammation can lead to a host of debilitating diseases such as rheumatoid arthritis, inflammatory bowel disease, asthma and chronic obstructive pulmonary disease, as well as acute processes such as sepsis. Therefore, cells must possess natural physiological mechanisms to terminate inflammation and limit tissue destruction.

Adenosine is an endogenous purine nucleoside that regulates numerous cellular functions including inflammation. Many different cell types produce and release adenosine during normal metabolic function. In pathological conditions such as inflammation, ischemia and hypoxia, adenosine production and release are elevated [3-5]. Endogenous adenosine is rapidly degraded and therefore its presence is limited to sites of inflammation. The biological activity of adenosine is mediated through specific membrane bound G protein-coupled receptors (GPCRs) termed P_1_-purinoceptors, also known as adenosine receptors (AR). To date four AR subtypes have been described [6]. Among them, A_2a_R is the most effective in down-regulating inflammation through modulation of intracellular cAMP levels [7,8]. A_2a_R is coupled to the stimulatory G protein Gαs [9]. Thus, adenosine A_2a_R occupation leads to an increase in intracellular cAMP levels. The anti-inflammatory role of A_2a_R has been confirmed by the observation that A_2a_R-deficient mice are hypersensitive to inflammatory stimuli [10].

GPCRs initiate signaling upon agonist binding to a site that has evolved to specifically recognize the agonist for the respective receptors, termed orthosteric binding sites. In addition, GPCRs also contain allosteric binding sites that are recognized by ligands and/or synthetic small molecules. These allosteric sites are topographically distinct from the orthosteric sites (for review see [11,12]). Therefore, structural features that determine ligand-binding to the allosteric sites are different from those of the orthosteric sites. Unlike the orthosteric ligands, allosteric ligands have little or no intrinsic ability to activate GPCRs upon engagement at the allosteric site. They modulate orthosteric ligand-mediated receptor function through conformational changes that result in altered affinity of orthosteric ligands for the receptors. Therefore, allosteric ligands can modulate and preserve the endogenous orthosteric ligand-mediated physiological responses, a property that has been successfully exploited for therapeutic benefit [13,14].

Activation of the A_2a_R using high affinity orthosteric ligands/agonists has demonstrated efficacy in a variety of animal models of inflammation. However, lack of in vivo AR subtype selectivity hindered their further development. We sought to enhance the responsiveness of A_2a_R to endogenously produced adenosine by using an allosteric ligand that positively modulates the receptor activity. Here we report the first evidence that endogenous adenosine-mediated A_2a_R activation can be enhanced using a positive allosteric modulator, AEA061 [15]. We show that positive allosteric modulation of the A_2a_R alters the K_d_ and B_max_ of the receptor with respect to the endogenous agonist, adenosine, causing increased agonist potency as well as efficacy, thereby enhancing functional responsiveness of the receptor.

## Methods

### Mice

Male BALB/cJ mice and A_2a_R null mice (C;129S-Adora2atm1Jfc/J; Jackson Laboratories) were housed at 68 – 72° F with a 12 h light/dark cycle, fed normal rodent chow and water ad libitum and were kept in a pathogen-free environment. A protocol approved by the Animal Care and Use Committee of the Molecular Medicine Research Institute was used in this study.

## Materials

NECA, CGS 21680, ZM 241385 and MRS 1754 were purchased from Tocris Biosciences. [^3^H]CGS 21680, GTPγ^35^S and WGA FlashPlates were purchased from PerkinElmer Corporation. LPS (E. coli O111:B4), growth media and all the reagents (unless otherwise stated) were purchased from Sigma-Aldrich.

### Cell culture

PC-12 cells (ATCC) were grown in DMEM supplemented with 5% FBS, 10% horse serum and amphotericin B (0.25 mg/ml). CHO-hA_2a_R cells were grown in DMEM/F-12 (1:1) supplemented with 10% FBS, 2 mM glutamine and G418 (0.2 mg/ml). All cells were maintained at 37°C in a 5% CO_2_ incubator.

### cAMP assay

PC-12 cells were washed with and resuspended in Hanks’ balanced salt solution (HBSS), transferred to 96-well plates (1.4×10^6^ cells per well) and incubated with rolipram (50 μM), adenosine, CGS 21680 and AEA061 at indicated concentration(s) for 15–30 min at 37°C. CHO cells expressing human A_2a_R (CHO-hA_2a_R; [16]) were seeded in the absence of G418 in 96-well plates (2×10^4^ cells/well) 20 h prior to assay. Assay conditions were essentially the same as above with the exception of inclusion of adenosine deaminase (ADA; 3U/ml) and adenosine 5′ [α,β-methylene] diphosphate (50 μM). Intracellular cAMP levels were quantified using Parameter ELISA kits (R&D Systems).

### Radioligand binding assay

The binding affinity of adenosine to hA_2a_R was evaluated using the [^3^H]-labeled A_2a_R-selective agonist CGS 21680. CHO-hA_2a_R cell membranes were prepared as described by Gao et al., [17]. Membranes (10 mg/well) in binding buffer (50 mM Tris pH 7.4, 10 mM MgCl_2_, 1 mM EDTA, 1.5 U/ml ADA) were incubated with AEA061 (10 μM) or vehicle (DMSO) in a 96-well WGA FlashPlate at 25°C for 15 min. After addition of [^3^H]CGS 21680 (0.0-110 nM), plates were incubated for an additional 75 min followed by washing with cold PBS containing AEA061 (10 μM) and [^3^H]CGS 21680. Binding was quantified using a TopCount scintillation counter. Specific binding was calculated by subtracting counts obtained in the presence of NECA (50 μM) from total counts.

### GTP binding assay

CHO-A_2a_R membranes were incubated in assay buffer (75 mM Tris (pH 7.5), 12.5 mM MgCl_2_, 1 mM EDTA, 100 mM NaCl, 3 μM GDP and 0.5 % BSA) containing CGS 21680 (0.0 – 1000 nM), ADA (2U/ml) and GTPγ^35^S (0.4 nM) with or without AEA061 (10 μM) in a 96-well WGA FlashPlate for 3 h at 25°C. Non-specific binding was estimated using GTP-γ-S (10 μM). Plates were washed twice with cold PBS and radioactivity was quantified using a TopCount scintillation counter.

### Cytokine assays

Human monocytes were isolated from buffy coat preparations (Stanford University blood bank) by depleting other cell types using MACS monocyte isolation kit II (Miltenyi Biotech) as suggested by the manufacturer. To isolate mouse splenocytes, spleens were aseptically removed, punctured and rinsed with RPMI 1640 medium to yield a cell suspension. Following red blood cell lysis with ACK buffer (Invitrogen Corporation), cells were seeded in 96-well plates in the same medium. Splenic monocytes/macrophages were isolated by plastic adherence after incubation for 2 h at 37°C and 5% CO_2_, followed by washing twice with PBS to remove nonadherent cells. Monocytes/macrophages were seeded in 96-well plates (2x10^5^ cells per well) and stimulated with phorbol 12-myristate 13-acetate (PMA; 5 ng/ml) or lipopolysaccharide (LPS; 5 – 50 ng/ml) in RPMI 1640 containing 1% FBS for 12 h in the presence of indicated concentration(s) of either AEA061 or NECA. For experiments evaluating the effects of A_2a_R antagonists, cells were pretreated for 15 min with the antagonist ZM 241385 at 37°C before stimulation with PMA. Splenocytes were seeded in 96-well plates (2×10^5^ cells per well) and stimulated with LPS (50 ng/ml) in DMEM containing 1% FBS for 18 h in the presence of indicated concentrations of AEA061. Cytokine/chemokine levels in the culture supernatants were quantified using Quantikine ELISA kits (R&D Systems) and/or bead-based multiplex immunoassays (Eve Technologies).

### Mouse model of endotoxemia

Male 8–10 weeks old A_2a_R null mice (C;129-Adora2atm1Jfc/J; Jackson laboratories) and receptor competent BALB/cJ control mice (Jackson laboratories; n = 10-14 per group) were dosed intravenously with AEA061 (0.1, 1 and 10 mg/kg) 10 min prior to i.p. injection of LPS (20 mg/kg). Two h after LPS dosing, mice were terminally bled under anesthesia using cardiac puncture technique. Plasma was isolated using lithium heparin tubes and stored at -80°C. Plasma cytokine/chemokine levels were quantified using Quantikine ELISA kits (R&D Systems) and/or bead-based multiplex immunoassays (Eve Technologies).

### Data analysis

Data were analyzed using GraphPad Prism Software. Dose response curves were generated by non-linear regression with a variable slope. Synergism between adenosine and AEA061 was analyzed using one-way ANOVA with the Bonferroni correction.

## Results

### Augmentation of adenosine-mediated cAMP production by PC-12 cells

**Figure 1:**
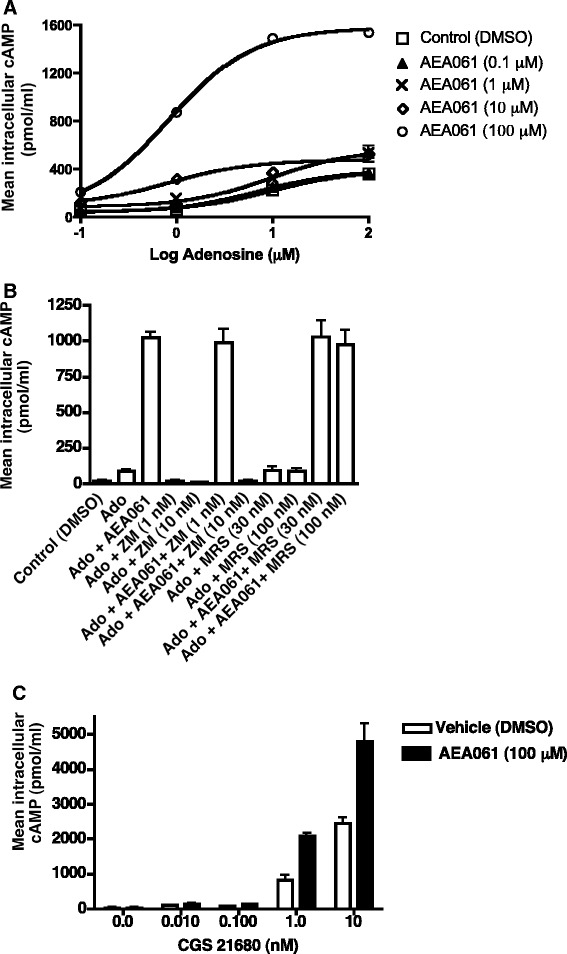
**Potentiation of agonist**-**mediated A**_2_ **R activation in PC**-**12 cells. (A)** AEA061 enhances adenosine-mediated cAMP production. PC-12 cells were incubated for 30 min with adenosine and AEA061. EC_50_ values in μM (with 95% confidence intervals in parentheses) were as follows: control = 9.7 (7.8 – 12.0); AEA061 (1 μM) = 9.5 (7.5 – 12.2); AEA061 (10 μM) = 0.84 (0.3 – 2.4); AEA061 (100 μM) = 0.83 (0.74 – 0.94). **(B)** AEA061-mediated potentiation of cAMP production is sensitive to ZM241385. PC-12 cells were pre-incubated with ZM241385 (ZM) and MRS 1754 (MRS) for 15 min prior to treatment with adenosine (Ado; 1 μM) and AEA061 (1 μM) for additional 30 min. **(C)** AEA061 enhances cAMP production mediated by CGS 21680. PC-12 cells were incubated for 15 min with AEA061 and CGS 21680. Mean cAMP levels ± SD of a representative experiment in triplicates are shown.

PC-12 cells, derived from a pheochromocytoma of rat adrenal medulla, are known to exhibit high Gαs-coupled AR activity. Agonist-mediated activation of the ARs in these cells leads to a significant rise in intracellular cAMP levels. We took advantage of this quantifiable cellular response to screen derivatives of a small molecule of plant origin for the ability to potentiate adenosine-mediated activation of endogenous ARs in intact PC-12 cells. To rule out the possibility that the elevated levels of cAMP were due to inhibition of phosphodiesterase 4 (PDE4) by the compounds, we utilized a saturating dose of a prototypical PDE4 inhibitor, rolipram. Using this screening paradigm, we have identified a family of non-agonist small molecules that modulate AR activity. One member of this family, AEA061 [15], enhanced adenosine-mediated cAMP production by PC-12 cells in a dose-dependent manner (Figure 1A). The synergism between AEA061 and adenosine was significant at 1 μM of the compound with adenosine levels ≥1 μM (p <0.05). Higher concentrations of AEA061 produced significant synergism at all concentrations of adenosine (p <0.05). On the basis of the EC_50_ values, AEA061 (10 μM) potentiated adenosine-mediated cAMP production by 11.5 fold. This AEA061-mediated shift in agonist EC_50_ was saturable, as 100 μM of AEA061 did not yield a greater effect. In addition, AEA061 increased the maximal response by 1.9, 2.4 and 4.2 fold at 1, 10 and 100 μM respectively. These results demonstrate that the affinity as well as the efficacy of adenosine at the endogenous Gαs-coupled adenosine receptors is enhanced by AEA061.

### Augmented cAMP production by PC-12 cells is mediated through the rat A_2a_R (rA_2a_R)

There are two Gαs-coupled adenosine receptor subtypes (A_2a_R and A_2b_R) whose activation leads to an increase in intracellular cAMP levels [18]. Although there are reports of A_2b_R expression by PC-12 cells, A_2a_R appears to be the predominant adenosine receptor subtype. To evaluate the role of A_2a_R and/or A_2b_R in AEA061-mediated enhancement of cAMP production, PC-12 cells were treated with the A_2a_R antagonist ZM 241385 and the A_2b_R antagonist MRS 1754. As shown in Figure 1B, adenosine increased cAMP production by 4.3 fold over the control and AEA061 (10 μM) enhanced cAMP levels by an additional 11.7 fold. As expected, ZM 241385 (1 nM and 10 nM) blocked adenosine-mediated cAMP production. In addition, ZM 241385 at 10 nM, but not 1 nM, blunted the adenosine plus AEA061-mediated cAMP production. In contrast, MRS 1754, an A_2b_R-specific antagonist, failed to inhibit adenosine- or adenosine plus AEA061-induced cAMP production. These results support the notion that enhanced-cAMP production by PC-12 cells in the presence of AEA061 is due to potentiation of rA_2a_R but not rA_2b_R activity.

To further investigate the observation that AEA061-dependent potentiation of cAMP production by PC-12 cells is due to enhanced-activation of the endogenous rA_2a_R, we evaluated the effects of AEA061 on cAMP production stimulated by the A_2a_R-selective adenosine analog CGS 21680. As shown in Figure 1C, CGS 21680 increased cAMP production in a dose-dependent fashion while the addition of AEA061 significantly enhanced it. Synergism between CGS 21680 and AEA061 was evident at 1 nM of CGS 21680 and above (p <0.05 – p <0.001), confirming our observation that the AEA061-dependent increase in intracellular cAMP levels is mediated through the endogenous rA_2a_R. Of note, AEA061, even at 100 μM, did not induce cAMP production in the absence of the agonist, indicating that the compound has no intrinsic activity and that AEA061-mediated enhancement of rA_2a_R activity is due to allosterism rather than agonism at the receptor.

### Enhancement of affinity and efficacy of adenosine at the human A_2a_ receptor (hA_2a_R)

**Figure 2:**
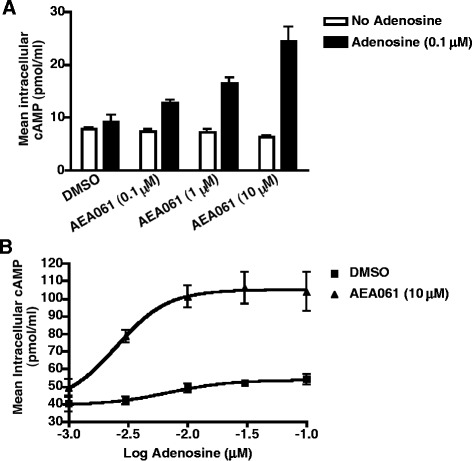
**Augmentation of adenosine**-**mediated activation of the human A**_**2a**_ **R** **(hA**_**2a**_ **R)** **. (A)** AEA061 enhances adenosine-mediated cAMP production by the hA_2a_R. CHO-hA_2a_R cells were incubated with adenosine, and AEA061 for 15 min. Mean cAMP levels ± SEM of a representative experiment is shown (n = 3). **(B)** AEA061 potentiates both the affinity and the efficacy of adenosine at the hA_2a_R. CHO-A_2a_R cells were incubated with AEA061 and varying concentrations of adenosine for 15 min. Mean EC_50_ values of three experiments with SEM were 4.4 ± 1.3 nM and 1.5 ± 0.49 nM without and with AEA061 respectively. Increase in E_max_ by AEA061 = 2.1 ± 0.27 fold.

Since the human A_2a_R (hA_2a_R) is the most pharmacologically characterized A_2a_R [19] relative to other species, we investigated the hA_2a_R for potential allosteric modulation. As A_2a_R is evolutionarily conserved in terms of sequence, structure and function, new knowledge gained here will serve as a guide to the A_2a_Rs of other species. To establish whether the hA_2a_R is amenable to functional enhancement, we evaluated the effects of AEA061 on cAMP production by CHO-hA_2a_R cells. As shown in Figure 2A, AEA061 dose-dependently increased cAMP production by these cells in the presence but not in the absence of adenosine. These results demonstrate that AEA061 has no intrinsic activity towards the hA_2a_R, but can allosterically augment agonist-mediated hA_2a_R activity. On the basis of EC_50_ values, AEA061 enhanced the adenosine-mediated response by 2.8 fold (Figure 2B). Moreover, AEA061 also increased the maximal response by 2.1 fold. These results establish that, similar to the rA_2a_R, positive allosteric modulation of the hA_2a_R causes enhanced adenosine potency and efficacy at the receptor.

### Potentiation of hA_2a_R-coupled Gα activation

**Figure 3:**
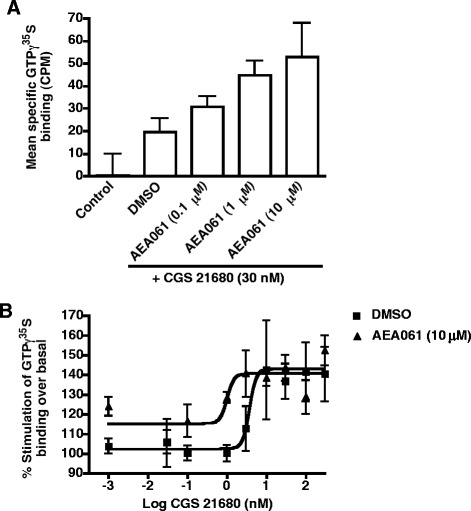
**Potentiation of hA**_**2a**_ **R**-**coupled Gα activation. (A)** AEA061 dose-dependently increases agonist-mediated GTPγ^35^S binding to membranes. CHO-hA_2a_R membranes were incubated with ADA, CGS 21680 and varying concentrations of AEA061. Each data point is the mean ± SEM of replicates of 5 determinations of a representative experiment. **(B)** AEA061 potentiates A_2a_R-mediated Gα activation. CHO-hA_2a_R membranes were incubated with ADA, AEA061 and varying concentrations of CGS 21680. Each data point is the mean ± SEM of replicates of 6 determinations of a representative experiment. EC_50_ values with 95% confidence intervals in parenthesis were as follows: control = 3.7 nM (2.195 - 6.294); AEA061 (10 μM) = 0.99 nM (0.7810 to 1.260).

The A_2a_R couples to two different classes of Gα subunits. A_2a_R coupling to the Gαs and Gαolf subunits that belong to the Gs class stimulates adenylate cyclase to increase intracellular cAMP upon agonist engagement at the receptor. In contrast, Gα15 and Gα16 subunits that belong to the Gq class couple the A_2a_R receptor to phospholipase C to stimulate the production of inositol phosphates upon receptor activation. A_2a_R-mediated activation of Gα subunits can be measured by incorporation of a radiolabeled non-hydrolysable GTP analog, GTPγ^35^S, into membrane preparations bearing the hA_2a_R. To investigate whether AEA061 enhances hA_2a_R-coupled Gα activation, agonist-induced increases in GTPγ^35^S incorporation were quantified in the presence or absence of the compound. As shown in Figure 3A, AEA061 dose-dependently enhanced CGS 21680-induced GTPγ^35^S incorporation. Analysis of the agonist dose response indicated that AEA061 potentiated agonist-mediated Gα activation by 3.7 fold. These results demonstrate that the responsiveness of agonist-mediated hA_2a_R-coupled Gα activation is enhanced by AEA061 and thus links AEA061 directly to A_2a_R activation.

### Enhancement of agonist binding to hA_2a_R

**Figure 4:**
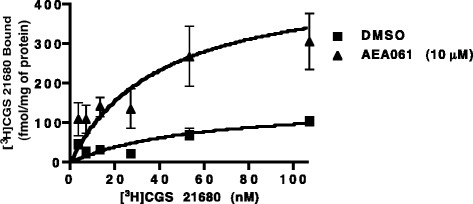
**Enhancement of agonist binding to hA**_**2a**_ **R.** Equilibrium binding of [^3^H]CGS 21680 to hA_2a_R in the presence of AEA061 (10 μM) and vehicle control is shown. CHO-hA_2a_R membranes were incubated with ADA and varying concentrations of [^3^H]CGS 21680 in the presence and absence of AEA061. B_max_ values: control = 152 fmol/mg; AEA061 (10 μM) = 470 fmol/mg. Values are mean ± SD of results of one representative experiment performed in triplicate.

In general, as a functional consequence of binding, allosteric modulators alter dissociation kinetics of the agonist. To investigate whether enhanced A_2a_R activation by AEA061 in the presence of agonist was due to altered agonist binding at the receptor, equilibrium binding studies were performed using the radiolabeled A_2a_R-selective agonist CGS 21680 and membrane preparations containing hA_2a_R. An enhancement in receptor affinity (1.8 fold) and B_max_ (3 fold) was observed with AEA061 (Figure 4). Taken together, these results demonstrate that AEA061 augments the affinity as well as the number of agonist binding sites at the hA_2a_R.

### Allosteric enhancement of the A_2a_R attenuates pro-inflammatory cytokine and chemokine production

**Figure 5:**
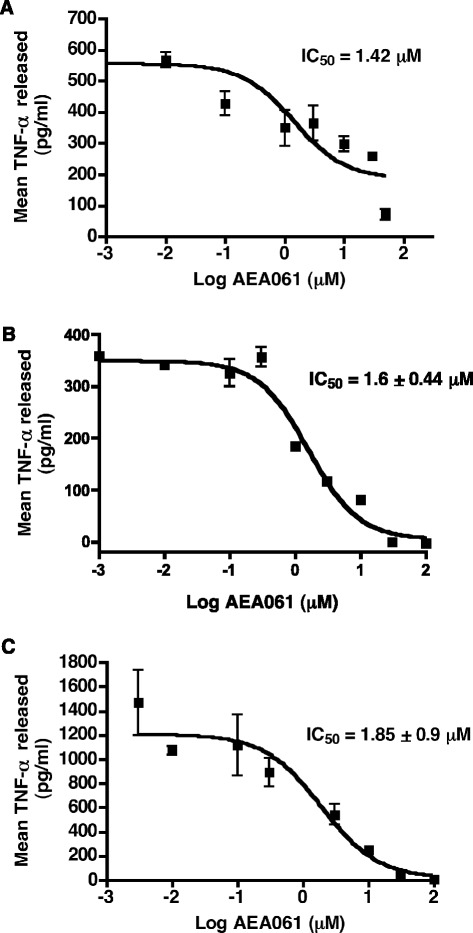
**Dose**-**dependent inhibition of LPS-** **and PMA-** **stimulated TNF**-**α production upon allosteric potentiation of the A**_**2A**_ **R.** Human monocytes were stimulated with LPS (5 ng/ml; **A**) or phorbol 12-myristate 13-acetate (PMA; 5 ng/ml; **C**) for 12 h whereas mouse monocytes were stimulated with LPS (50 ng/ml; **B**) for 18 h in the presence of varying concentrations of AEA061. TNF-α in the culture medium was quantified as described in Materials and Methods. Values and error bars represent means and SEMs of one of three representative experiments performed in triplicate.

**Figure 6:**
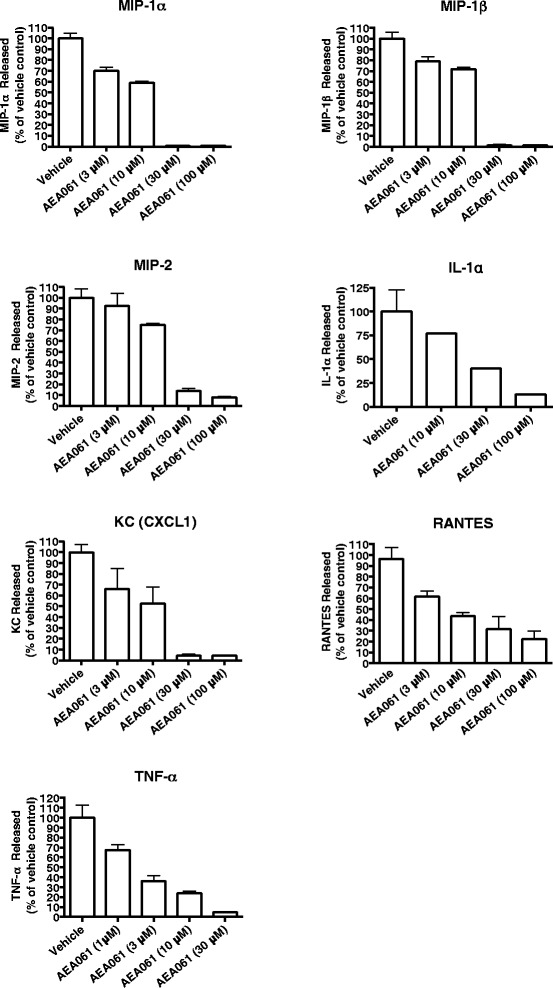
**Inhibition of cytokine and chemokine production by splenocytes upon allosteric enhancement of the A**_**2A**_ **R.** Mouse splenocytes were stimulated with LPS (50 ng/ml) for 18 h in the presence of the indicated concentrations of AEA061. Cytokines/chemokines in the culture medium were quantified as described in Material and Methods. Values are mean ± SEM of the results of one representative experiment performed in triplicate.

Activation of A_2a_R has been shown to inhibit TNF-α production by monocytes/macrophages [20-22]. We therefore investigated the effect of allosteric enhancement of A_2a_R activity on TNF-α production mediated by two different stimuli acting through two distinct signaling pathways: stimulation of the TLR4 receptor by LPS, and protein kinase C (PKC)/mitogen-activated protein kinase (MAPK) activation by PMA. Consistent with the role of allosteric enhancement of the hA_2a_R, AEA061 inhibited TLR4-mediated TNF-α production by freshly-isolated human and mouse monocytes in a dose-dependent manner with IC_50_ values of 1.42 μM (Figure 5A) and 1.6 ± 0.44 μM (Figure 5B) respectively. AEA061 also dose-dependently inhibited TNF-α production mediated by PKC/MAPK activation in freshly isolated human monocytes (IC_50_ = 1.85 ± 0.9 μM, Figure 5C). In addition, we evaluated the effects of A_2a_R positive allosteric modulation on the production of cytokines and chemokines by LPS-stimulated splenocytes. Of interest, the production and/or release of KC, IL-1α, MIP-1α, MIP-1β, MIP-2, RANTES and TNF-α were consistently inhibited by allosteric enhancement of the A_2a_R in a dose-dependent manner (Figure 6) while additional cytokines and chemokines remained unchanged (data not shown). A standard battery of preclinical safety pharmacology tests on AEA061 did not reveal any toxicity or side effects, hence ruling out cytotoxicity as a possible cause for the inhibition of cytokine/chemokine production. Taken together, these results suggest that positive allosteric modulation of the A_2a_R of monocytes and splenocytes is sufficient to enhance receptor responsiveness to endogenous adenosine and inhibit production of multiple pro-inflammatory cytokines and chemokines.

### A_2a_R antagonism dampens the allosteric enhancement-mediated inhibition of TNF-α production by monocytes

**Figure 7:**
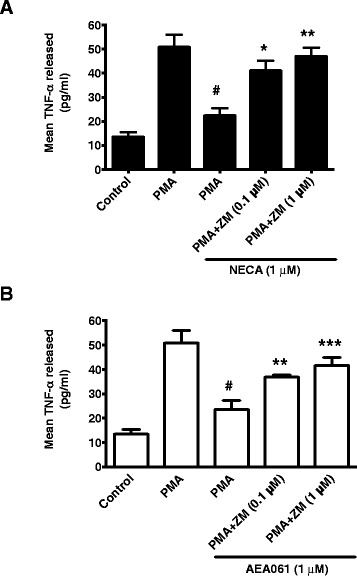
**Reversal of AEA061**-**mediated inhibition of TNF-** **α production by ZM 241385.** Human monocytes were pretreated for 15 min with the A_2A_R antagonist ZM 241385 before PMA stimulation (5 ng/ml) and incubation with either the AR agonist NECA (1 μM; **A**) or AEA061 (1 μM; **B**) for 12 h. TNF-α in the culture medium was quantified by ELISA. # = p <0.001 PMA versus PMA plus AEA061 or NECA; * = p <0.05, ** = p <0.01 *** = p <0.001 PMA plus AEA061 or NECA with ZM versus without ZM.

To examine whether the inhibition of TNF-α production by AEA061 is mediated through selective allosteric enhancement of the A_2a_R, we evaluated the effects of AEA061 in the presence and absence of the A_2a_R-specific antagonist, ZM 241385, on TNF-α production by human monocytes. As a control, we used the non-selective high affinity AR agonist, NECA, in parallel experiments. NECA inhibited PMA-stimulated TNF-α production by freshly isolated human monocytes/macrophages and the A_2a_R-selective antagonist, ZM 241385, reversed the NECA-mediated inhibition in a dose-dependent manner (Figure 7A). Similarly, ZM 241385 blunted the inhibition of PMA-stimulated TNF-α production enhanced by AEA061 in a dose-dependent manner (Figure 7B). The A_2a_R antagonist, ZM 241385, at 1 μM restored PMA-stimulated TNF-α responsiveness in both NECA- and AEA061-treated cells. These results support the notion that selective allosteric enhancement of the A_2a_R of monocytes renders them more responsive to endogenous adenosine thereby further enhancing adenosine’s anti-inflammatory activity.

### Allosteric enhancement of the A_2a_R alters circulating plasma levels of pro-inflammatory and anti-inflammatory cytokines in endotoxemic A_2a_R+/+ mice but not in A_2a_R −/− mice

**Figure 8:**
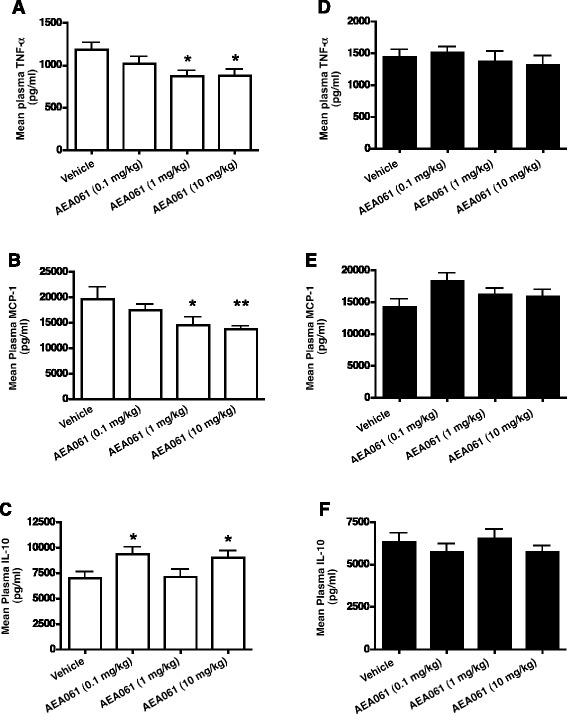
**Effects of allosteric enhancement on circulating plasma cytokines and chemokines in the LPS-** **induced mouse model of endotoxemia.** Study groups consisted of male age matched control BALB/cJ mice (open bars; **A-** **C**) and A_2A_R-deficient mice (closed bars; **D-** **F**). Animals were administered either vehicle or AEA061 (0.1, 1 and 10 mg/kg) iv 10 min prior to ip injection of LPS (20 mg/kg). Plasma cytokine/chemokine levels were quantified 2 h after LPS dosing as described in Materials and Methods. Mean plasma cytokine/chemokine levels ± SEMs of 10–14 animals per treatment group are indicated. Statistical difference between the vehicle and the treatment groups were determined by one-way ANOVA; *, p <0.05 and **, p <0.01.

To further investigate the notion that allosteric enhancement significantly augments endogenous adenosine-mediated A_2a_R activation and subsequent inhibition of inflammation in vivo, we utilized a mouse model of endotoxemia. A_2a_R null mice and receptor competent BALB/cJ control mice were injected intraperitoneally with LPS, and after 2 h plasma was collected for the measurement of cytokine and chemokine levels. In LPS-stimulated receptor competent BALB/cJ mice, allosteric enhancement of the A_2a_R with AEA061 at 1 and 10 mg/kg significantly reduced plasma TNF-α and MCP-1 levels compared to vehicle treatment (Figure 8A and B). Although AEA061 administration reduced plasma levels of IL-12, p40, MIP1-α, MIP-2, MIG and M-CSF relative to that of the untreated animals, the level of reduction did not reach statistical significance (data not shown). In contrast, AEA061 significantly enhanced the plasma levels of the anti-inflammatory cytokine, IL-10, at 0.1 and 10 mg/kg (Figure 8C). These results demonstrate that positive allosteric modulation of the A_2a_R enhances receptor responsiveness to endogenous adenosine and down-regulates the inflammatory cytokine cascade in vivo. The group of A_2a_R null mice that received vehicle alone had higher circulating plasma TNF-α (Figure 8D) and lower MCP-1 (Figure 8E) and IL-10 (Figure 8F) levels relative to their BALB/cJ receptor-competent counterparts. AEA061 at any concentration did not alter plasma levels of TNF-α, MCP-1 or IL-10 in LPS-treated A_2a_R null mice. These results confirm that selective allosteric enhancement of the A_2a_R by AEA061 is responsible for the anti-inflammatory effects observed in vivo. It is unclear why AEA061 enhanced IL-10 production at 0.1 and 10 kg/ml in receptor competent BALB/cJ mice yet failed to enhance IL-10 production at 1 mg/kg while the same dose of 1 mg/kg significantly affected TNF-α and MCP-1 production. More in depth studies are needed to clarify this observation.

## Discussion

We demonstrate that AEA061-mediated augmentation of A_2a_R activity bears the hallmarks of positive allosteric modulation. First, both the rA_2a_R and the hA_2a_R were unresponsive to AEA061 in the absence of the agonist. Second, affinity and maximal response of the rA_2a_R and the hA_2a_R were enhanced by AEA061. Third, hA_2a_R-coupled Gα activation was augmented by AEA061. Lastly, affinity as well as the number of agonist binding sites at the hA_2a_R were enhanced by AEA061.

The observation that positive allosteric modulator (PAM), AEA061, increases the number of binding sites can be explained by equilibrium receptor binding kinetics. Agonist binding stabilizes the signaling competent active conformation of the A_2a_R and shifts the equilibrium away from the inactive conformation. AEA061, stabilizes the agonist-bound conformation of the A_2a_R and strengthens this equilibrium shift further. As this assay utilizes membranes, the increase in AEA061-mediated agonist binding cannot be explained by an increase in receptor number.

Enhancement of agonist binding at the A_2a_R has been reported by two groups [23,24]. Bruns and Lu reported an increase in agonist binding to rat striatal membranes when treated with the synthetic small molecule PD10918 [23]. However, the functional consequence of this enhanced agonist binding was not investigated. In a more recent study using a substituted 8-azaadenine derivative, Georgi et al. [24] reported augmented agonist binding at the human receptor as well as increased agonist-mediated relaxation of rat aortic rings ― observations which were attributed to allosteric modulation. Further studies are necessary, however, to fully support the postulated mechanism.

The anti-inflammatory role of the A_2a_R has been well documented. Activation of the A_2a_R by high affinity orthosteric agonists reduces the production of pro-inflammatory cytokines (TNF-α, IL-6, IL-12) and chemokines (MCP-1, MIP-1, MIP-2) and increases the production of the anti-inflammatory cytokine (IL-10) in vitro (for review see [25]). Consistent with these effects, enhancement of A_2a_R activation with AEA061 inhibits TNF-α production by LPS- and PMA-stimulated monocytes/macrophages as well as MIP-1α, MIP-1β and MCP-2 production by LPS-stimulated splenocytes. However, we did not see a consistent in vitro effect on the production of IL-6, IL-12 and IL-10 upon A_2a_R allosteric enhancement at 12 h. This may be due to the time-dependent nature of cytokine/chemokine expression and the need to evaluate cytokines/chemokines at earlier time points in order to fully understand the effects of positive allosteric enhancement of the A_2a_R on immunomodulation.

Allosteric modulators have no intrinsic activity on the receptors. In order to highlight the absolute requirement for adenosine for AEA061-mediated A_2a_R potentiation, in the cell-based cAMP assay, cells were pretreated with adenosine deaminase and adenosine 5′ [α,β-methylene] diphosphate to rid the cells of adenosine and to inhibit further production of extracellular adenosine respectively. In the monocyte/macrophage/splenocyte cytokine production assays, however, exogenous addition of adenosine is not needed to generate AEA061-mediated enhancement of A_2a_R activation and subsequent inhibition of pro-inflammatory cytokine production, as adenosine is produced both extracellularly and intracellularly by many different cell types including monocytes/macrophages and splenocytes during their normal cell metabolism.

In vivo activation of the A_2a_R also leads to a reduction in pro-inflammatory cytokines and an increase in anti-inflammatory cytokines levels (for review see [25]). These effects explain the observed efficacy of A_2a_R orthosteric agonists across a variety of animal models of inflammatory disease. In a murine model of sepsis, the high affinity orthosteric agonist, ATL313, decreases circulating plasma levels of pro-inflammatory cytokines and chemokines such as TNF-α, MIP-1α, MCP-1 [26] and increases the plasma level of the anti-inflammatory cytokine IL-10 [26] within 4 h of LPS injection. Consistent with this observation, we found that positive allosteric modulation of the A_2a_R upon LPS challenge reduces significantly plasma levels of TNF-α and MCP-1 and increases IL-10. Although we observed a reduction in MIP-1α, the level of reduction did not reach statistical significance.

Comparison of in vitro and in vivo anti-inflammatory effects of AEA061 indicates a 10-fold reduction in vivo efficacy with respect to inhibition of TNF-α production. One can envision several possibilities for this lack of one-to-one correlation between in vitro and in vivo efficacy of AEA061. First, it may stem from inherent properties of AEA061 such as plasma binding, metabolism and tissue distribution that can reduce its in vivo availability. Second, in vitro effects of AEA061 are reflective of the impact of A_2a_R potentiation on inflammatory cytokine production by one cell type in isolation. In vivo effects of AEA061 on inflammatory cytokine production, however, captures a collective response generated by multiple immune cells under the influence of variety of intracellular interactions. Third, allosteric enhancement of the A_2a_R by AEA061 is adenosine dependent. Adenosine is rapidly degraded in vivo by adenosine deaminase, thus has a short half-life (less than 10 seconds). Hence, in vivo, receptor enhancement may be dictated by the endogenous adenosine and not by AEA061 levels. It is possible that in vivo, adenosine levels do not rise to the same levels as in vitro, and that this may lead to less inhibition of inflammatory cytokines in vivo.

In summary, we have demonstrated that the anti-inflammatory effects of AEA061 are due solely to positive allosteric modulation of the A_2a_R. First, through extensive in vitro characterization we established that AEA061 is a PAM of the A_2a_R. Second, we have shown that pharmacological blockade of the A_2a_R with a selective antagonist abolished AEA061-mediated inhibition of TNF-α production. Third, AEA061 did not inhibit plasma pro-inflammatory cytokines/chemokines levels nor did it increase plasma anti-inflammatory cytokine levels in A_2a_R-deficient mice. Collectively, our data support the notion that A_2a_R activation can be enhanced using a PAM and is sufficient to decrease systemic inflammation. A PAM, such as AEA061, can thus harness the inherent anti-inflammatory and immunomodulatory effects of adenosine by enhancing the responsiveness of the A_2a_R to endogenously produced adenosine.

In sepsis, reduced A_2a_R responsiveness to endogenous adenosine as characterized by decreased receptor affinity to adenosine is, at least in part, responsible for hyperactivation of polymorphonuclear leukocytes (PMNs) [27,28]. The enhanced release of reactive oxygen species by hyperactivated PMNs is a key step in the pathogenesis of sepsis. Similarly it is likely that monocytes/macrophages also exhibit reduced A_2a_R responsiveness and that allosteric enhancement of the A_2a_R with AEA061 may have restored adenosine responsiveness of the A_2a_R on monocytes/macrophages in our endotoxemia model.

**Figure 9:**
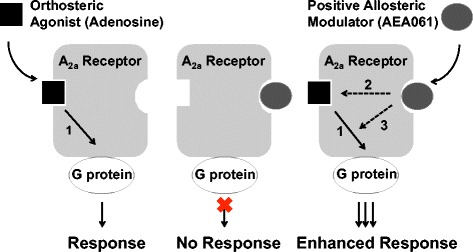
**Proposed mechanism of positive allosteric modulation of A**_**2a**_ **R function by AEA061.** The orthosteric agonist (adenosine) and the positive allosteric modulator, AEA061, bind to topographically distinct sites at the A_2A_R. Unlike adenosine, AEA061 on its own has no effect on A_2A_R response. However, it can enhance affinity and efficacy of adenosine and hence the receptor response. 1. Orthosteric (adenosine) agonism; 2. Allosteric (AEA061) affinity (potency) enhancement; 3. allosteric (AEA061) efficacy signaling (capacity) enhancement.

To the best of our knowledge, the present study is the first comprehensive demonstration of positive allosteric modulation of the A_2a_R. Because of its anti-inflammatory and immunomodulatory role, the A_2a_R has been a therapeutic target for a number of disease conditions characterized by inflammation such as sepsis, ischemia, rheumatoid arthritis, and wound healing [25,29]. Selective orthosteric agonism at the A_2a_R as a therapeutic strategy has been extensively studied using high affinity agonists for over three decades [30,31]. The therapeutic utility of high affinity orthosteric agonists is hindered, however, by the lack of adenosine receptor subtype selectivity upon systemic exposure [32,33] as well as inappropriate on-target receptor activation. In contrast, allosteric enhancement of A_2a_Rs may provide an improved specificity and safety profile. Specificity is enhanced since a PAM binds to a site that is distinctly different from the orthosteric agonist (adenosine)-binding site (Figure 9) and is unlikely to be conserved among different AR subtypes; hence allosteric modulation is less likely to produce undesired effects owing to non-selectivity. Enhancement of safety may be anticipated since a PAM will potentiate adenosine-mediated receptor activity but have no intrinsic agonist activity (Figure 9). Thus, a PAM potentiates and preserves the temporal and spatial distribution of the adenosine-mediated physiological response. This approach is conceivably superior to others from a therapeutic and safety perspective as it focuses the therapeutic benefit at the site of disease, does not employ exogenous orthosteric agonists and preserves the natural pattern of A_2a_R activation by endogenous adenosine while enhancing receptor responsiveness.

## Conclusions

Using an allosteric modulator, AEA061, we demonstrate that the A_2a_R is amenable to allosteric enhancement. Increasing A_2a_R responsiveness to endogenous adenosine with the administration of a positive allosteric modulator, that has no intrinsic ability to activate the A_2a_R, can effectively attenuate inflammation in vivo. Hence, allosteric enhancement of the A_2a_R presents a novel, disease site-focused, therapeutic strategy to reduce progression of disease characterized by inflammation.

## Abbreviations

AR: Adenosine receptor
A_2a_R: Adenosine A_2a_ receptor
PDE4: Phosphodiesterase 4
cAMP: Cyclic adenosine monophosphate
CGS 21680: 4-[2-[[6-Amino-9-(N-ethyl-β-D-ribofuranuronamidosyl)-9H-purin-2-yl]amino]ethyl] benzenepropanoic acid hydrochloride
DMSO: Dimethyl sulfoxide
ELISA: Enzyme-linked immunosorbent assay
GPCR: G protein-coupled receptor
MRS 1754: N-(4-Cyanophenyl)-2-[4-(2,3,6,7-tetrahydro-2,6-dioxo-1,3-dipropyl-1H-purin-8-yl)phenoxy]-acetamide
NECA: 5′-N-Ethylcarboxamidoadenosine
PAM: Positive allosteric modulator
PBS: Phosphate buffered saline
SD: Standard deviation
PMA: Phorbol 12-myristate 13-acetate
SEM: Standard error of the mean
ZM 241385: 4-(2-[7-Amino-2-(2-furyl)[1,2,4]triazolo[2,3-a][1,3,5]triazin-5-ylamino]ethyl)phenol
